# Comparison of Umbilical Cord Mesenchymal Stem Cells and Fibroblasts as Donor Nuclei for Handmade Cloning in Sheep Using a Single-Cell Transcriptome

**DOI:** 10.3390/ani14040589

**Published:** 2024-02-10

**Authors:** Weijian Li, Yalan Liu, Guizhen Zhou, Zhuo Li, Zhen Wang, Li Wang, Xiuling Ma, Xuguang Wang

**Affiliations:** 1 College of Animal Science, Xinjiang Agricultural University, Urumqi 830052, China; weijian_lee1028@163.com (W.L.); yalan_0117@163.com (Y.L.); lizhuoxnd@163.com (Z.L.); wangzhen156324@163.com (Z.W.); 13565549026@163.com (L.W.); mxlde_123456@163.com (X.M.); 2 College of Animal Science and Technology, China Agricultural University, Beijing 100091, China; guizhen_orchid@126.com

**Keywords:** single-cell transcriptome, umbilical cord mesenchymal stem cells, sheep, handmade cloning, nuclear transplantation

## Abstract

**Simple Summary:**

There have been few studies on the use of umbilical cord mesenchymal stem cells as donor nuclei in nuclear transplantation, despite their advantages of less reprogramming difficulty and shorter reprogramming distance. This study compared differences at the chromatin level, the level of differentially expressed genes, the level of histone modifications, and the level of DNA methylation in eight-cell embryos after using umbilical cord mesenchymal stem cells and fibroblasts as nucleus donors, using a single-cell transcriptome.

**Abstract:**

Oocytes are efficient at reprogramming terminally differentiated cells to a totipotent state. Nuclear transfer techniques can exploit this property to produce cloned animals. However, the overall efficiency is low. The use of umbilical cord mesenchymal stem cells (UC-MSCs) as donor nuclei may increase blastocyst rates, but the exact reasons for this remain unexplored. A single-cell transcriptomic approach was used to map the transcriptome profiles of eight-cell embryos that were in vitro-fertilized and handmade-cloned using umbilical cord mesenchymal stem cells and fibroblasts as nuclear donors. Differences were examined at the chromatin level, the level of differentially expressed genes, the level of histone modifications and the level of DNA methylation. This research provides critical information regarding the use of UC-MSCs as a preferred donor nucleus for nuclear transfer techniques. It also offers unique insights into the mechanism of cellular reprogramming.

## 1. Introduction

The cytoplasm of oocytes has the ability to reprogram the chromatin state of terminally differentiated cells to a totipotent state [1]. This ability can be exploited to generate cloned progeny or nuclear transferred embryonic stem cells (ntESCs) using nuclear transfer techniques. Handmade cloning (HMC) is a more convenient and faster method of nuclear transfer that does not require a micromanipulator [2]. It has a higher throughput and yield than traditional cloning methods [3,4]. A variety of animals have been handmade-cloned to produce offspring [5]. Therefore, handmade cloning plays an important role in the production of genetically modified animals [6,7] and the conservation of rare species [8,9].

Currently, cloning technology still faces some challenges that must be addressed before it can be widely implemented. One of the main obstacles is the issue of low cloning efficiency. While the cloning efficiency of cattle can reach 1–20% [10,11], the overall cloning efficiency of most species, including mice, is only 1–10% [12,13].

The efficiency of cloning is influenced by two main factors: the reprogramming capacity of the cytoplasm and the ease of reprogramming the donor chromatin. Several methods have been developed to improve cytoplasmic reprogramming, including overexpression of KDM4D [14], overexpression of DUX [15], and modification of imprinted genes [16,17]. And the main approach in the exploration of the ease of reprogramming of donor nucleus chromatin is to find cell lines with shorter reprogramming pathways and less difficult reprogramming. Umbilical cord mesenchymal stem cells (UC-MSCs) are multipotent stem cells derived from the fetal umbilical cord. They have the advantages of faster proliferation, shorter reprogramming pathway, and less difficulty in reprogramming [18,19]. Previous studies have demonstrated that using UC-MSCs as donor nuclei in bovine clones can increase the blastocyst rate compared to fibroblasts and can successfully establish pregnancy [20]. However, the use of UC-MSCs as donor nuclei is still limited, and the mechanism behind the enhanced blastocyst rate is not yet understood.

This study utilized a single-cell transcriptomic approach to map the transcriptome profiles of eight-cell embryos that were in vitro-fertilized and handmade-cloned using UC-MSCs and fibroblasts as nuclear donors. The study explored differences at the chromatin level, the level of differentially expressed genes, the level of histone modifications, and the level of DNA methylation. This research provides important information regarding the use of UC-MSCs as a preferred donor nucleus for nuclear transfer techniques, and also provides some unique clues for the study of cell reprogramming mechanisms.

## 2. Materials and Methods

Unless otherwise stated, all chemicals were purchased from Sigma Chemical Co., St. Louis, MO, USA. All plasticware used in this study was purchased from Corning Incorporated, Corning, NY, USA.

### 2.1. In Vitro Maturation of Oocytes

The sheep ovaries were obtained from the slaughterhouse and returned to the laboratory within three hours. Follicles of 2–6 mm on the ovarian surface were extracted using a 10 mL syringe with a 1.2 mm diameter needle after cleaning. Under a stereoscope, COCs with homogeneous cytoplasm and at least three layers of granulosa cells were selected and placed into an IVM solution. The solution contained 1 IU/mL FSH, 1 IU/mL LH, 1 μg/mL E_2_, 0.1 mmol/L *L*-cysteine, and 10% FBS dissolved in TCM-199. The COCs were cultured for in vitro maturation at 38.5 °C in 5% CO_2_ and saturated humidity.

### 2.2. Preparation of Donor Cells

Sheep umbilical cord MSCs and sheep ear fibroblasts, obtained from 2-month-old sheep, were thawed one to two weeks prior to the start of the experiment. They were then cultured in DMEM/F12 medium containing 10% FBS. For cloning assays, trypsin digestion was performed for three minutes and terminated by adding two times the volume of PBS containing 2% FBS. The cells were then washed twice by centrifugation at 100× *g* for five minutes. Subsequently, the cells were immersed in TCM-199 solution with 2% FBS (T2 solution) and left at room temperature.

### 2.3. De-Nucleation and Fusion

When oocytes matured for 20 h, those containing the first polar body were selected and set aside after removing the granulosa cells using 1 mg/mL of hyaluronidase. Oocytes that contained the first polar body were treated with T2 solution containing 1 mg/mL pronase. Then, 1/3 of the cytoplasm on the first polar body side of the oocyte was cut off using a cutting knife in T2 solution containing 2.5 μg/mL Cytochalasin B. The remaining 2/3 of the cytoplasm was singled out and recovered in the T2 solution. Once all oocytes containing the first polar body were cut, the fusion operation was carried out. A small number of cells were aspirated and placed in T2 prior to fusion. At the start of the fusion process, the cytoplasm was treated with a T2 solution containing 0.5 mg/mL phytohemagglutinin for 3 to 5 s. Subsequently, smooth-shaped, medium-sized, and refractive somatic cells adhered to form cytoplasmic–somatic–cytoplasmic pairs. The pairs were then transferred into the fusion solution, which consisted of 300 mmol/L mannitol and 1 mg/mL polyvinyl alcohol dissolved in pure water. The pairs were neatly aligned on the electrodes at the end of the 0.5 mm fusion groove using an alternating current of 0.114 kV/cm. The direct current parameter of the fusion apparatus was set to 1.8 kV/cm, *n* = 1 for fusion operation. Following fusion, the cytoplasmic–somatic–cytoplasmic pairs were immersed in T2 solution for 1 h. Spherical reconstructed embryos, in which the somatic cells had been incorporated into the oocyte cytoplasm and had a good morphology, were selected for activation after all fusion operations had been completed.

### 2.4. In Vitro Fertilization of Oocytes

At 23 h of maturation, the oocytes were washed with an in vitro fertilization solution, which was synthetic oviductal fluid containing 4 mg/mL BSA, 100 μg/mL heparin, and 1 mmol/L glutamine. Subsequently, they were transferred to a droplet of in vitro fertilization solution to await fertilization. Spermatozoa were obtained from the epididymis of slaughtered animals. The epididymis was cut to a size of 5 cm × 5 cm using scissors and then immersed in the in vitro fertilization solution, followed by incubation at 38.5 °C for 5 min to allow the spermatozoa to swim out. Only sperm with a density greater than 1 × 10^7^ and a viability greater than 0.85 were used. The spermatozoa were centrifuged twice at 100× *g* and then added to a drop of the in vitro fertilization solution containing oocytes for in vitro fertilization. The fertilization process was carried out in embryos at 38.5 °C, 5% CO_2_, and saturated humidity conditions for 18 h.

### 2.5. Activation and In Vitro Culture

To activate the nuclear transfer embryos, the selected reconstituted embryos were placed in a T2 solution containing 5 μmol/L ionomycin for 5 min. They were then transferred to a T2 solution containing 2 mmol/L 6-dimethylaminopurine for 3–5 h. For in vitro culture, the embryos were cultured in a medium containing essential and non-essential amino acids, 4 mg/mL BSA, and 1 mmol/L glutamine in synthetic oviductal fluid. The culture was maintained at 38.5 °C and 5% CO_2_ under saturated humidity for 24 h to observe the cleavage rate and for 7 days (168 h) to observe the blastocyst rate.

### 2.6. RNA Isolation, Library Preparation, and Sequencing

RNA was extracted from the 8-cell embryos fertilized and cloned using UC-MSC and fibroblasts (the three groups were labelled IVF, DFUC and DFF, respectively), and embryos were lysed using a single-cell lysis kit (Invitrogen, Carlsbad, CA, USA) according to the manufacturer’s instructions. Subsequently, the 1st cDNA was obtained using a single-cell reverse transcription kit (Invitrogen, Carlsbad, CA, USA) according to the manufacturer’s manual and then amplified using PCR with the 1st cDNA as a template. The cDNA product was purified using Ampure XP beads and its concentration was measured with a Qubit^®^ 3.0 Fluorometer (Thermo, Waltham, MA, USA). The integrity and distribution of fragments were assessed using an Agilent 2100 Bioanalyzer and an Agilent 2100 High Sensitivity DNA Assay Kit (Agilent, Santa Clara, CA, USA). Library construction was performed by taking 40 ng of product from each sample of satisfactory quality and breaking it into 350 bp fragments using ultrasound. The fragments then underwent end repair, addition of base A, addition of an adapter, and PCR amplification. Finally, Novaseq S2 (Illumina, San Diego, CA, USA) was used for paired-end sequencing.

### 2.7. Quality Control and Mapping

The study obtained raw data and processed them using Fastp (v0.23.4) to remove the adapter, reads containing excessive N, and reads with low average quality values. Q30 and GC contents were then calculated for the clean data, which were used for all subsequent analyses. HISAT2 (v2.2.1) was used to compare the clean reads for each sample to the sheep reference genome (ARS-UI_Ramb_v3.0, 20 July 2023), and QuliMap (v2.2.2) was used to calculate read distributions.

### 2.8. Quantification and Differential Expression Analysis

Quantification was performed using the featureCounts function in the R package Rsubread (v2.14.2). The count values were then normalized to TPM values for subsequent analysis. Differential expression analysis was conducted using the R package DESeq2 (v1.42.0). The screening criteria for differentially expressed genes were |log2 fold change| ≥ 1 and *p* adjust ≤ 0.05.

### 2.9. KEGG, GO, and GSEA Enrichment Analyses

The DAVID website “https://david.ncifcrf.gov (accessed on 20 December 2023)” was used to analyze KEGG and GO, followed by plotting using the R package ggplot2 (v3.4.4). Gene Set Enrichment Analysis (GSEA) was performed using the R package clusterProfiler (v4.10.0). DEGs were considered significantly enriched at *p* < 0.05 for all enrichment analyses.

### 2.10. Real-Time Quantitative PCR (RT-qPCR)

The cloned and in vitro-fertilized embryos underwent 1st cDNA synthesis using the SuperScript™ IV CellsDirect™ cDNA Synthesis Kit (Invitrogen, Carlsbad, CA, USA) following the manufacturer’s instructions. Briefly, 8-cell embryos were lysed using SuperScript™ IV CellsDirect™ Lysis Solution, Lysis Enhancer, and DNase I. Lysis was terminated by the addition of SuperScript™ IV CellsDirect™ Stop Solution. SuperScript™ IV RT Master Mix was used for 1st cDNA synthesis at 25 °C for 10 min, 50 °C for 10 min, and 85 °C for 5 min. RT-qPCR was performed using Bio-Red CFX 96 at 95 °C for 15 min, followed by 40 cycles of 95 °C for 10 s and 60 °C for 32 s. The relative expression was calculated using the 2^−ΔΔCt^ method, with β-actin serving as the internal reference gene (Table 1).

### 2.11. Statistical Analysis

Statistical analyses were conducted using SPSS 26.0 software. *t*-tests were used for the analyses, and significance was considered at *p* < 0.05. The symbol * indicates *p* < 0.05, ** indicates *p* < 0.01, and ns indicates *p* > 0.05.

## 3. Results

### 3.1. Transcriptome Profiles

**Figure 1:**
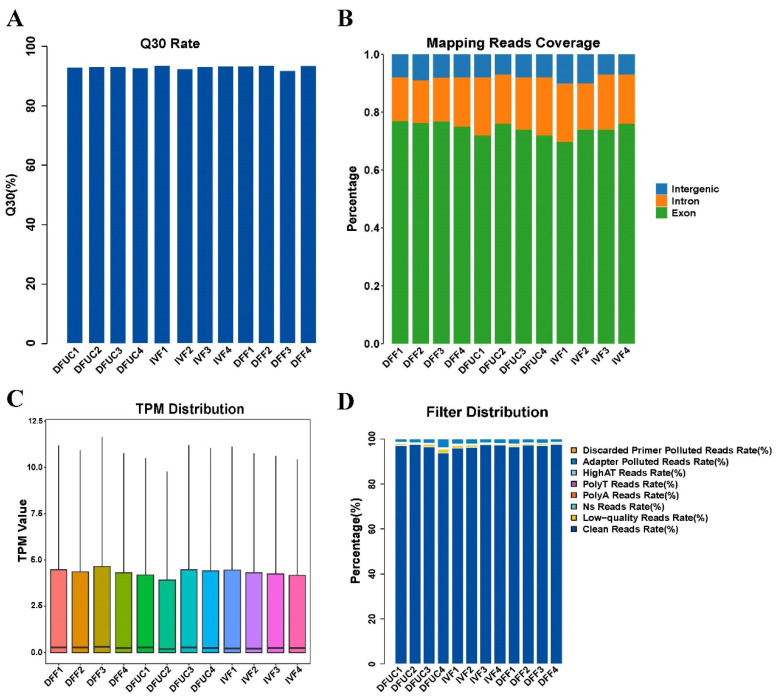
RNA-seq base quality and comparison statistics: (**A**) Q30 rate of base measured for each sample. (**B**) Proportion of reads mapped to exon, intron, and intergenic regions. (**C**) Distribution of TPM for each sample. (**D**) Filtering statistics for reads.

The study consisted of three groups (DFF, DFUC, IVF), each with four replicates, resulting in a total of 12 samples. The average number of reads per sample was 41.6 million. The unique mapping rates were all above 95%, while the multi-locus mapping rates ranged from 4.9% to 6.7% (Table 2). The Q30 rates were all above 90%, and the clean read rates were all above 95%. Additionally, the clean reads mapped to exon regions were all above 70% (Figure 1A,B,D).

**Figure 2:**
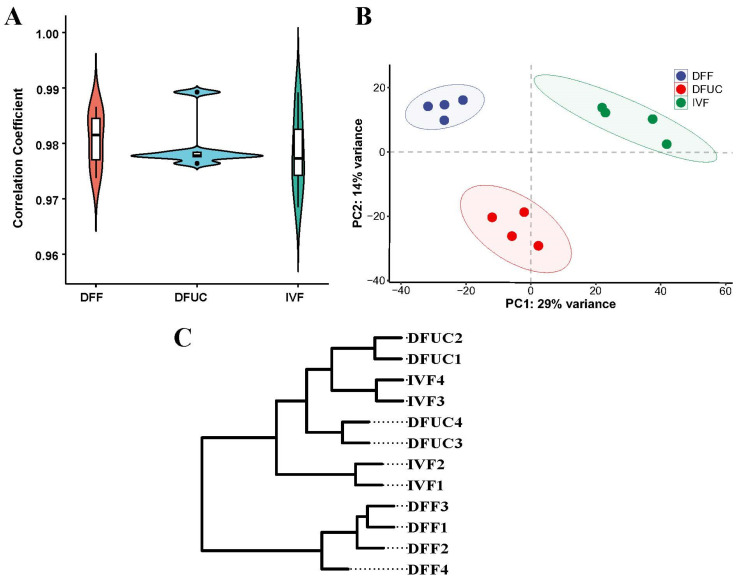
RNA-seq inter- and intra-group correlation analysis: (**A**) Intra-group correlation violin plot. (**B**) PCA analysis plot. (**C**) Hierarchical clustering plot.

Pearson correlation analysis showed a correlation of over 95% between the samples. PCA clustering indicated that the samples in the DFF, DFUC, and IVF groups could be clustered together, respectively. However, when all the samples were mapped to PC1, the DFF and IVF clusters were on opposite sides, while the DFUC cluster was in the middle and closer to the IVF cluster. This suggests that the UC-MSCs as donor nuclei could be closer to the level of IVF (Figure 2B). A hierarchical cluster analysis was conducted, revealing that the 12 samples were divided into two clusters. DFF was found to be alone in one cluster, while DFUC and IVF were clustered together in the other. This suggests that DFUC and IVF are more similar.

The results show that the RNA-seq reads from all 12 samples had high rates of clean reads, comparison, and Q30. Furthermore, the use of UC-MSCs as donor nuclei resulted in RNA profiles that were closer to IVF than DFF.

### 3.2. Analysis of DFF, DFUC, and IVF Differentially Expressed Genes

**Figure 3:**
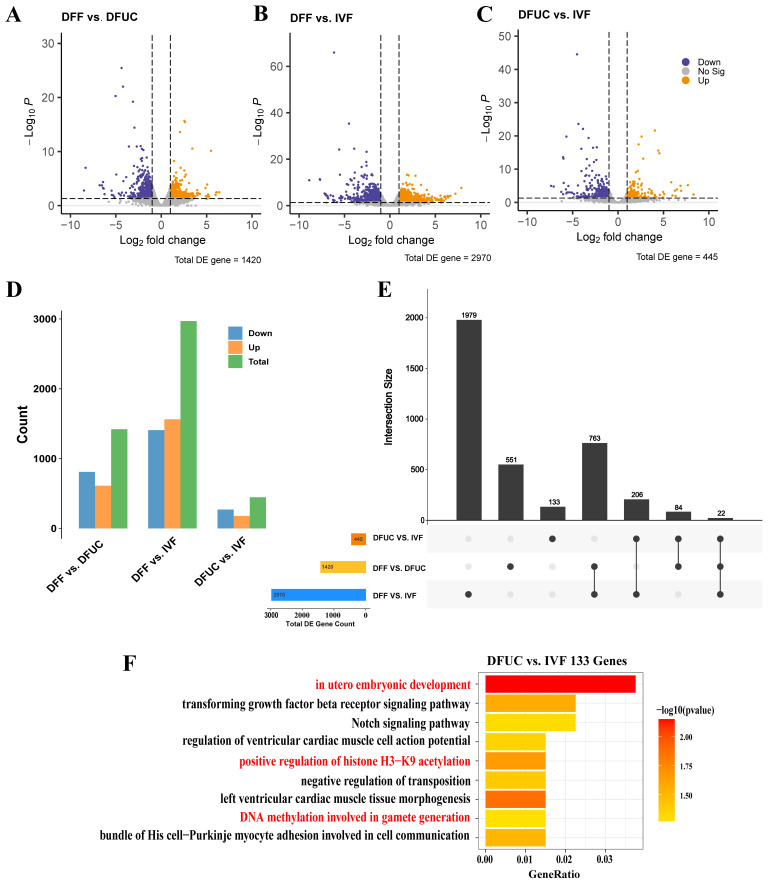
Statistical analysis of differentially expressed genes: (**A**–**C**) Volcano plot of differentially expressed genes. (**D**) Statistical histogram of differentially expressed genes between samples. (**E**) Upset plot of differentially expressed genes. (**F**) GO enrichment analysis of 133 specific differentially expressed genes in DFUC vs. IVF.

Although the expression levels of most genes were similar in all samples (Figure 1C), there were still differentially expressed genes in each group (Figure 3A–C). There were 1420 differentially expressed genes in DFF vs. DFUC and 2970 differentially expressed genes in DFF vs. IVF, but surprisingly there were only 445 differentially expressed genes in the DFUC vs. IVF groups (Figure 3D). The analysis using Upset plots revealed that there were 133 genes unique to DFUC vs. IVF, but 1979 genes unique to DFF vs. IVF, indicating that UC-MSCs were reprogrammed to a greater extent as donor nuclei and more closely resembled IVF embryos compared to fibroblasts. Furthermore, the analysis identified 206 genes that overlapped with differentially expressed genes in DFUC vs. IVF and DFF vs. IVF. Using GO enrichment analysis of 133 differentially expressed genes specific to DFUC vs. IVF revealed that their functions were mainly enriched in in utero embryonic development, the transforming growth factor-beta receptor signaling pathway, positive regulation of histone H3-K9 acetylation, DNA methylation involved in gametogenesis, and the Notch signaling pathway.

The results indicate that although UC-MSCs as donor nuclei produced reconstructed embryos that were closer to the level of IVF, 445 genes were still not successfully reprogrammed, and 133 of these 445 genes were DFUC-specific genes that were not reprogrammed. These 133 genes were found to be associated with the histone H3K9 locus, DNA methylation, TGFβ, and Notch signaling pathways.

### 3.3. GO, KEGG, and GSEA Enrichment Analyses

**Figure 4:**
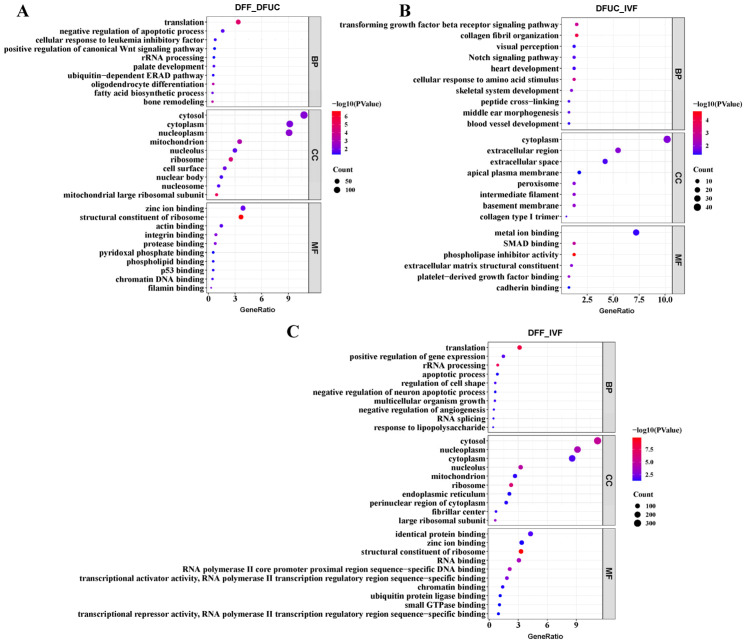
GO enrichment analyses: (**A**–**C**) indicate the bubble plots of GO enrichment analyses for DFF vs. DFUC, DFUC vs. IVF, and DFF vs. IVF, respectively.

GO enrichment analysis showed that DFF vs. DFUC differentially expressed genes were mainly enriched in Biological Process (BP) terms including translation, negative regulation of apoptotic process, cellular response to leukemia inhibitory factor, and positive regulation of canonical Wnt pathway. The Cellular Component (CC) terms were mainly enriched in the cytosol, cytoplasm, nucleoplasm, and mitochondrion. The Molecular Function (MF) category is mainly enriched in zinc ion binding, structural constituents of ribosomes, and actin binding (Figure 4A). Comparing DFUC and IVF, differentially expressed genes in BP were mainly enriched in the transforming growth factor-beta receptor signaling pathway, collagen fibril organization, visual perception, and the Notch signaling pathway, and differentially expressed genes in CC were mainly enriched in the cytoplasm, extracellular region, extracellular space, and apical plasma membrane. The MF category is primarily enriched in metal ion binding, SMAD binding, and phospholipase inhibitor activity (Figure 4B). In the comparison between DFF and IVF, the differentially expressed genes were mainly enriched in BP categories including translation, positive regulation of gene expression, rRNA processing, and apoptotic process. The differentially expressed genes in the CC categories were mainly enriched in the cytosol, nucleoplasm, cytoplasm, nucleolus, and mitochondrion, and MF categories were mainly enriched in identical protein binding, zinc ion binding, and structural component of ribosome (Figure 4C).

**Figure 5:**
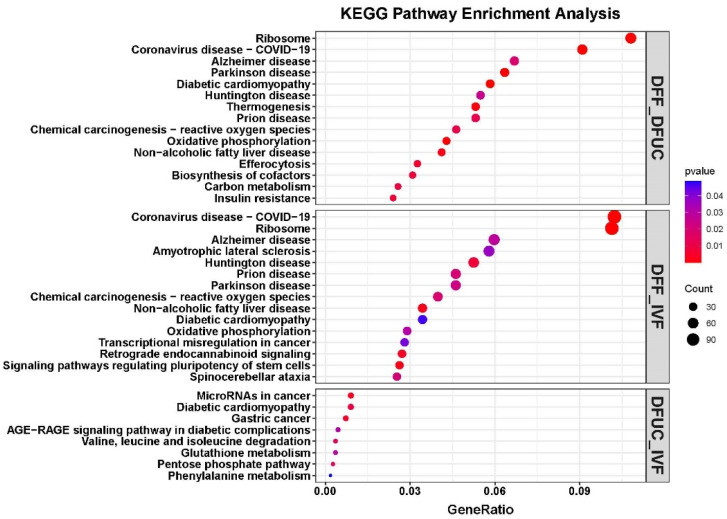
DFF vs. DFUC, DFUC vs. IVF, DFF vs. IVF KEGG enrichment analysis bubble plot, respectively.

The KEGG enrichment analysis revealed that DFF vs. DFUC was primarily enriched in ribosome; coronavirus disease—COVID-19; Alzheimer’s disease; and Parkinson’s disease. Meanwhile, DFF vs. IVF was mainly enriched in coronavirus disease—COVID-19; ribosome; Alzheimer’s disease; and amyotrophic lateral sclerosis. Lastly, DFUC vs. IVF was mainly enriched in MicroRNAs in cancer, diabetic cardiomyopathy, and gastric cancer (Figure 5).

**Figure 6:**
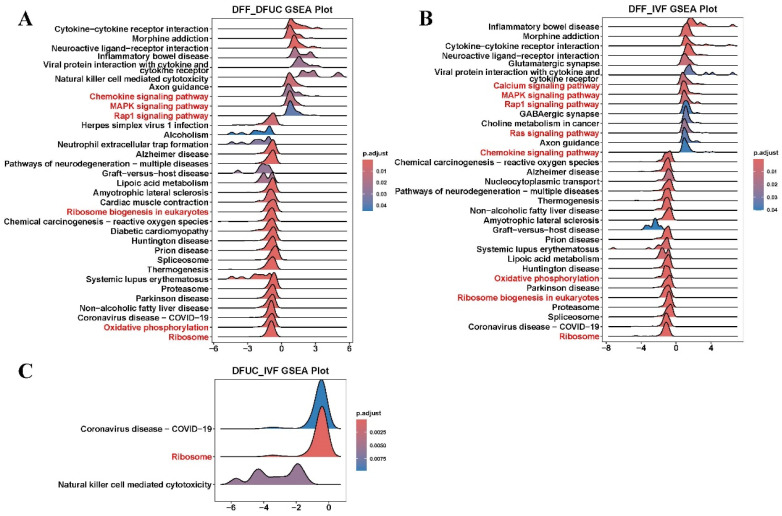
GSEA enrichment analysis ridge diagrams: (**A**–**C**) indicate the GSEA enrichment analysis ridge diagrams for DFF vs. DFUC, DFUC vs. IVF, and DFF vs. IVF, respectively.

**Figure 7:**
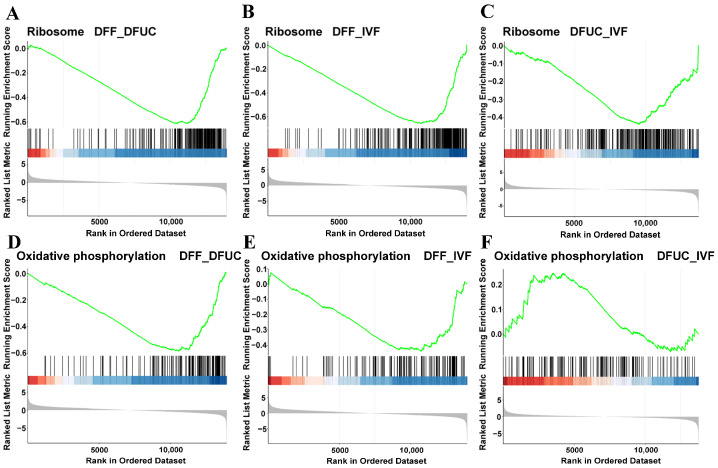
GSEA enrichment analysis results: (**A**–**C**) indicate GSEA enrichment analysis results of the ribosome pathway for DFF vs. DFUC, DFUC vs. IVF, and DFF vs. IVF. (**D**–**F**) indicate GSEA enrichment analysis results of the oxidative phosphorylation pathway for DFF vs. DFUC, DFUC vs. IVF, and DFF vs. IVF.

The GSEA enrichment analysis showed that the chemokine, MAPK, and Rap1 signaling pathways were highly expressed in DFF vs. DFUC. In contrast, the ribosome biogenesis in eukaryotes, oxidative phosphorylation, and ribosome-related pathways were expressed at a low level. In the comparison between DFF and IVF, the calcium signaling pathway, MAPK signaling pathway, and Rap1 signaling pathway were highly expressed, while the oxidative phosphorylation, ribosome biogenesis in eukaryotes, and ribosome-related pathways were expressed at a low level. Lastly, a low expression of ribosomes can be found in DFUC vs. IVF. In addition to the pathways mentioned above, the GSEA results indicate that the use of fibroblasts as a donor nucleus results in abnormalities in several disease pathways, including Alzheimer’s disease and inflammatory bowel disease, when compared to UC-MSCs as a donor nucleus (Figure 6). In combination with previous GO and KEGG enrichment analyses, it is evident that DFUC vs. IVF, DFUC vs. DFUC, and DFUC vs. IVF all exhibit abnormalities in the ribosome or oxidative phosphorylation pathways. By plotting the results of GSEA enrichment analysis of individual pathways, it is evident that the ribosome pathway is suppressed during the development of SCNT. This is indicated by the concentration of most genes of DFUC vs. IVF, DFF vs. DFUC, and DFF vs. IVF at the ribosome pathway in the low-expression region (Figure 7A–C). In the oxidative phosphorylation pathway, gene aggregation is present in the low expression region of DFF vs. DFUC and DFF vs. IVF, but there is no obvious aggregation of DFUC vs. IVF genes (Figure 7D–F).

The results show that the ribosome pathway is expressed at a low level regardless of whether UC-MSCs or fibroblasts are used as donor nuclei. However, using UC-MSCs as donor nuclei was successful in rescuing and restoring the normal level of the oxidative phosphorylation pathway. Additionally, using UC-MSCs as donor nuclei reduced or even rescued the aberrant expression occurring in many disease pathways compared to fibroblasts.

### 3.4. Heatmap and Protein Interaction Network Analysis (PPI)

**Figure 8:**
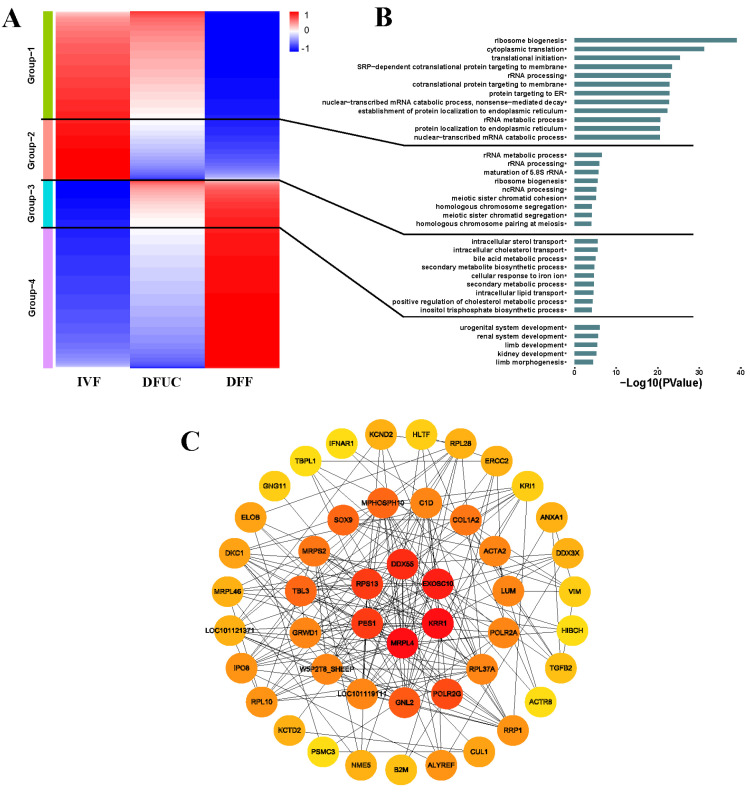
Heatmap and PPI network analysis: (**A**) indicates the heatmap of differentially expressed genes of IVF, DFUC, and DFF. (**B**) indicates the results of the respective GO enrichment analysis of each group in the heatmap. (**C**) Analysis of Group 2–3 PPI network in the heatmap.

The heatmap of the up- and down-regulated differentially expressed genes showed that UC-MSCs, as donor nuclei, were able to rescue 69.7% of the reprogrammed aberrant genes (Group 1 and Group 4). However, 30.3% of the genes were not rescued (Group 2 and Group 3). Of the unrescued genes, 16.9% were abnormally reprogrammed down-regulated genes (Group 2), and 13.3% were abnormally reprogrammed up-regulated genes (Group 3) (Figure 8A). The GO enrichment analysis showed that the genes in Group 1 of the rescued region were mainly enriched in some biological processes, including ribosome biogenesis, cytoplasmic translation, translational initiation, and protein targeting to the endoplasmic reticulum. Meanwhile, the genes in Group 4 were primarily enriched for developmental processes related to the urogenital and renal systems, as well as limb and kidney development. The down-regulated genes in Group 2, which were aberrantly reprogrammed in the unrescued regions, were mainly enriched in biological processes such as the rRNA metabolic process, meiotic sister chromatid cohesion, homologous chromosome segregation, and meiotic sister chromatid segregation. On the other hand, Group 3 was enriched in up-regulated genes that were aberrantly reprogrammed, specifically those involved in intracellular sterol transport, secondary metabolic processes, and intracellular lipid transport (Figure 8B). Furthermore, we conducted a PPI network analysis to identify six HUB genes that are related to ribosome and RNA metabolism. These genes, namely *DDX55*, *EXOSC10*, *KRR1*, *MRPL4*, *PES1*, and *RPS13*, were found among the abnormally reprogrammed up- and down-regulated genes (Figure 8C).

The results indicate that UC-MSCs can be used as donor nuclei to successfully reprogram 69.7% of genes. However, 30.3% of the genes could not be rescued, and these genes were mainly related to intracellular ribosome metabolism, chromosome segregation, and macromolecule metabolism. Abnormal reprogramming regions were mainly related to ribosome and RNA metabolisms, as revealed by the PPI network analysis.

### 3.5. Chromosome-Level Differential Expression Analysis

**Figure 9:**
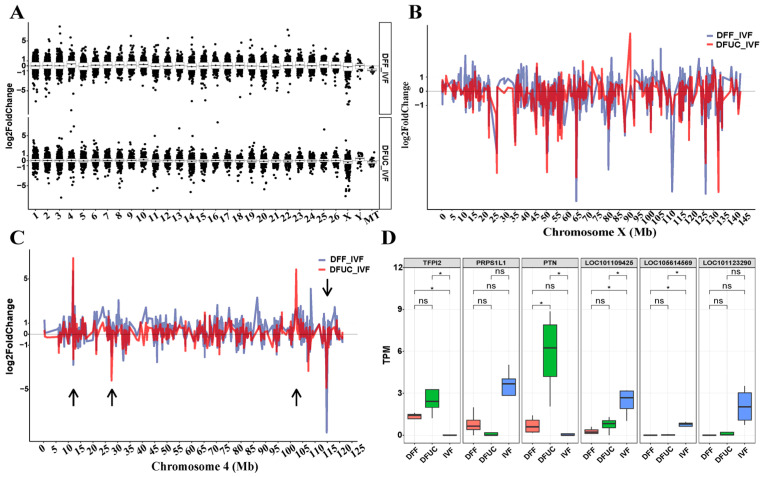
Chromosome-level differential expression analysis: (**A**) Graph indicating the expression level points of each chromosome. (**B**) Graph of the expression level lines of chromosome X. (**C**) Graph of the expression level lines of chromosome 4. (**D**) Graph of the major gene box patterns of the four differentially expressed regions of chromosome 4. The symbol * indicates *p* < 0.05, and ns indicates *p* > 0.05.

Sheep have 27 pairs of chromosomes, consisting of 26 autosomes and 1 sex chromosome. The nuclear transfer technique takes all the chromosomes from the donor nucleus, which means that the level of reprogramming at the chromatin level of the donor nucleus has a major influence on the development of the nuclear transfer embryo. When comparing the expression levels of DFUC vs. IVF and DFUC vs. IVF at the chromosome level, it is evident that the average expression level of DFUC vs. IVF on autosomes did not undergo a significant skew. However, there was a significant decrease in the expression levels of DFUC vs. IVF on the X chromosome and MT gene, while there was a significant increase in expression levels on the Y chromosome (Figure 9A). The comparison between DFF vs. IVF revealed varying degrees of skewed expression in both autosomes and sex chromosomes. Chromosome 4 showed a significant up-regulation of expression, while the X chromosome and MT gene showed a considerable down-regulation of expression (Figure 9A). The expression line plot of chromosome 4 indicates the presence of four distinct clusters of abnormal expression (Figure 9C, As shown by the arrow). The analysis of the genome browser showed that the four clusters of abnormal expression were primarily caused by six genes. Box plots of the expression of these six genes indicated that, compared to IVF, *TFPI2* was significantly overexpressed in both donor nuclei (*p* < 0.05), while *PTN* was significantly overexpressed in UC-MSCs as donor nuclei (*p* < 0.05). *LOC10119425* and *LOC105614569* were found to be significantly underexpressed in both donor nuclei (*p* < 0.05) (Figure 9D). Furthermore, the fold plot of X chromosome expression indicates a significant decrease in expression levels in both donor nuclei (Figure 9B).

The results indicate that UC-MSCs can perform sufficient levels of reprogramming and normalize the expression levels of most genes compared to fibroblasts in terms of autosomal expression levels. However, neither of the two donor nuclei could perform sufficient reprogramming in the sex chromosomes and MT genes.

### 3.6. Analysis of Histone Methylation and Acetylation and DNA Methylation Gene Expression Patterns and Validation of Transcriptome Validity Using RT-qPCR

**Figure 10:**
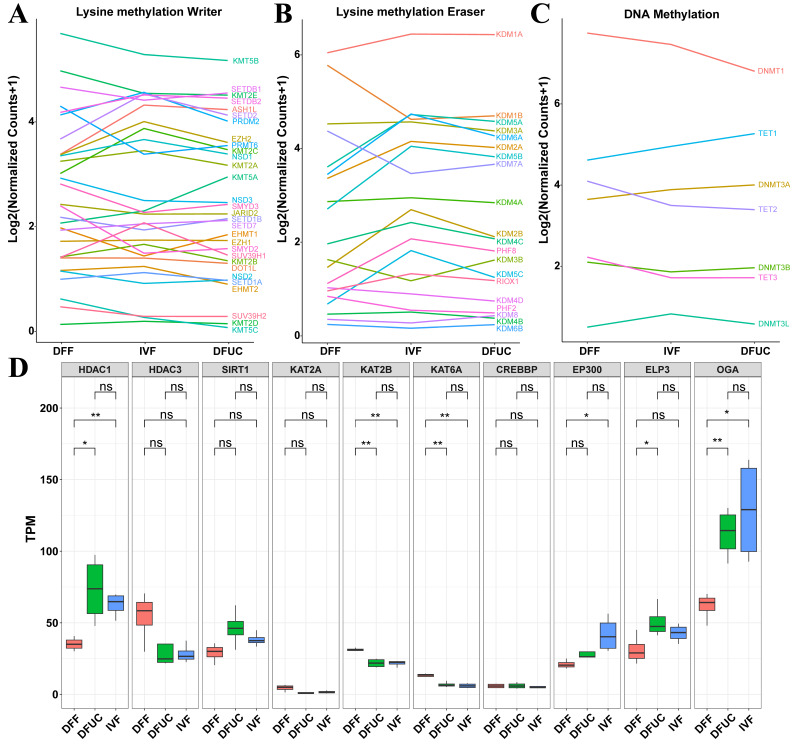
Analysis of histone methylation and acetylation with DNA methylation and demethylation gene expression patterns: (**A**–**C**) Each indicates a graph of changes in the expression of lysine methylase writer, lysine methylase eraser, and DNA methylation-related enzymes. (**D**) indicates box plots of the expression of several major histone acetylases and deacetylases. The symbol * indicates *p* < 0.05, ** indicates *p* < 0.01, and ns indicates *p* > 0.05.

**Figure 11:**
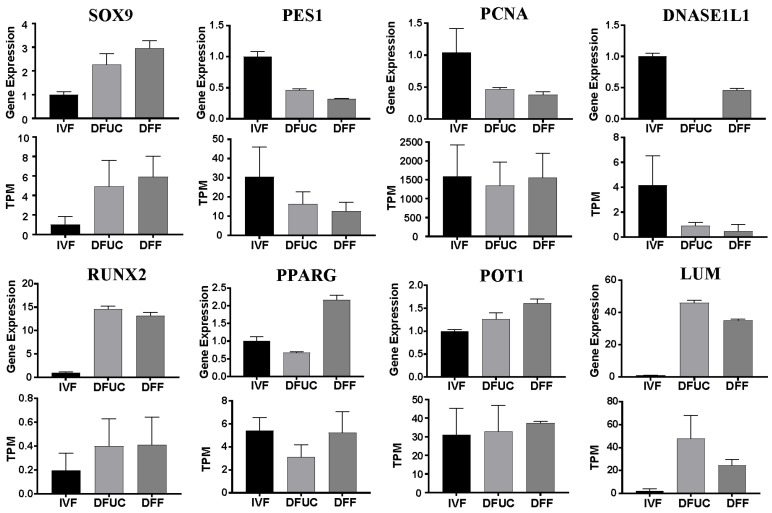
Validation of RNA-seq using RT-qPCR.

The Venn diagram revealed 133 genes that were non-reprogrammed by UC-MSCs as donor-nucleus-specific and were associated with the H3K9 locus and DNA methylation (Figure 3E). Subsequently, we investigated whether other genes related to histone modification also exhibited abnormal reprogramming. The results showed that many lysine methylation writers, lysine methylation erasers, DNA methylation writers, and DNA methylation erasers of the reconstructed embryos obtained with fibroblasts as the donor nucleus showed abnormal reprogramming (Figure 10). However, many enzyme activities were successfully restored by using UC-MSCs as donor nuclei. Despite this, aberrantly expressed genes such as *SUV39H1*, *KMT5A*, *EZH2*, *KDM5C*, and *KDM6A* were still present. In other words, the H3K27, H3K4, H3K9, and H4K20 loci remained abnormal, as shown in Figure 10A,B. Regarding DNA methylation, *DNMT1* exhibited low expression while *TET1* exhibited high expression after using UC-MSCs as donor nuclei, indicating an overall bias towards demethylation (Figure 10C). There was no significant difference in the expression of either acetylases or deacetylases between the IVF group and the handmade cloning group after using UC-MSCs as the nucleus donor (*p* > 0.05) in terms of histone acetylation. However, a significant abnormality was observed in the expression of the broad-spectrum deacetylase *HDAC1* and the broad-spectrum acetylases *KAT2B*, *KAT6A*, and *EP300* after using fibroblasts as the nucleus donor (*p* < 0.05) (Figure 10D). Finally, eight genes were selected at random and the validity of the transcriptome data was verified using RT-qPCR. The results indicated that the transcriptome data and RT-qPCR data were consistent (Figure 11).

The results indicate that using UC-MSCs as donor nuclei can rescue many writers and erasers in terms of lysine methylation modification. However, abnormalities still exist at the H3K27, H3K4, H3K9, and H4K20 sites. Regarding DNA methylation, *DNMT1* expression was low, while *TET1* expression was high, indicating a more favorable chromatin open state for the ZGA process. Furthermore, the level of histone acetylation in UC-MSCs used as the donor nucleus was closer to that of IVF.

## 4. Discussion

In recent years, most approaches to improving cloning efficiency have focused on how to improve the cytoplasmic reprogramming ability during the ZGA period [10,11], but there are few reports on exploring the difficulty of nucleus-donor reprogramming. Preliminary experiments conducted in our laboratory have shown that the blastocyst rate of nucleus-donor fibroblasts was approximately 20%. However, when using UC-MSCs as nucleus donors, the blastocyst rate was able to reach 40–50% and successfully gestate the recipient ewes. Lee et al. [21] discovered that using fetal fibroblasts as a nucleus donor resulted in a 25.6% blastocyst rate in porcine clones, while using spermatogonial stem cells as a nucleus donor resulted in a higher blastocyst rate of 47.8%. Similarly, Ps et al. [22,23] achieved a blastocyst rate of 19% using fibroblasts as nucleus-donor blastocysts in Indian buffalo clones, but were able to increase the rate to 29% by using amniotic fluid or amniotic MSCs as the nucleus donor. However, the mechanism by which adult stem cells can increase cloning efficiency remains unclear.

This study analyzed the transcriptome profiles of fibroblasts, UC-MSCs as nucleus donor 8–16-cell cloned embryos, and in vitro-fertilized 8–16-cell embryos using a single-cell transcriptome. PCA analysis showed that the samples clustered into three groups. The cloned embryos with UC-MSCs as nucleus donors were positioned between the cloned embryos with fibroblasts as nucleus donors and IVF embryos when mapped to PC1 (Figure 2B). Hierarchical clustering indicated that the cloned embryos with UC-MSCs as nucleus donors clustered with IVF (Figure 2C). Regarding differentially expressed genes, there were 2970 in the cloned embryos with fibroblasts as the nucleus donor compared to IVF, while only 445 were found in the cloned embryos with UC-MSCs as the nucleus donor compared to IVF embryos (Figure 3A–C). Under the same conditions, the overall expression profiles of cloned embryos with UC-MSCs as nuclei donors were more similar to those of IVF embryos. On the other hand, UC-MSCs were found to be more easily reprogrammed compared to fibroblasts. An analysis of the 445 differentially expressed genes between cloned embryos with UC-MSCs as nuclei donors and IVF embryos revealed that 133 of them were specific differentially expressed genes. These genes function mainly in the regulation of the H3K9 locus and DNA methylation, which is consistent with previous findings that found the reprogramming blocking region to be enriched by H3K9me3 [14]. Furthermore, it has been discovered that a high expression of the demethylase KDM4D/E of H3K9me3 significantly improves cloning efficiency and birth rate in various animals [24,25,26]. It is yet to be determined whether the overall efficiency can be synergistically enhanced by using UC-MSCs as nucleus donors followed by a high expression of KDM4D/E.

Next, KEGG, GO, and GSEA enrichment analyses were conducted. The results revealed that cloned embryos with UC-MSCs as nucleus donors exhibited abnormalities primarily in the TGFβ, Notch, and ribosome pathways compared to IVF embryos. Cloned embryos using fibroblasts as nucleus donors had abnormalities mainly in the ribosome, oxidative phosphorylation, and calcium compared to IVF embryos. Additionally, MAPK and other related pathways were found to be abnormal. Placental abnormalities contribute to the low efficiency and high miscarriage rate of the nuclear transfer technique. Previous studies have shown that the TGFβ pathway is associated with placental abnormalities in bovine nuclear transfer embryos [27]. It is yet to be determined whether TGFβ leads to placental abnormalities in sheep nuclear transfer. On the other hand, this study demonstrates that the expression of Sirt1 in UC-MSCs as nucleus donor embryos was slightly higher than that in IVF embryos (Figure 10D). Additionally, Sirt1 can act as an inhibitor of Notch on cell differentiation [28]. However, further investigation is required to determine the specific function and mechanism of the Notch pathway in cloning and cell reprogramming techniques. Oxidative phosphorylation primarily occurs within mitochondria. Mitochondrial abnormalities have been observed in bovine, sheep, and porcine embryos produced by nuclear transplantation [29,30,31,32]. However, the use of UC-MSCs as donor nuclei may partially rescue the abnormality of oxidative phosphorylation (Figure 7E,F). The exact mechanism requires further research.

Heatmapping of the differentially expressed genes showed that the use of UC-MSCs as donor nuclei rescued approximately 70% of the reprogrammed abnormal genes compared to fibroblasts as donor nuclei (Figure 8A). The analysis revealed that these genes were primarily associated with translation and organ development. This suggests that using UC-MSCs as donor nuclei may be more advantageous for fetal development after pregnancy. However, it remains to be seen whether UC-MSCs as donor nuclei can achieve a higher clone birth rate, which requires further verification over a longer period. While UC-MSCs can rescue most of the reprogrammed abnormal genes when used as donor nuclei, there remains a 30% portion of genes that cannot be rescued. This subset of genes is primarily associated with chromosome segregation and intracellular macromolecular substance metabolism. Chromosome segregation and centromere abnormalities are common issues in cloning technology [33,34,35], and further exploration is needed to overcome or optimize them in sheep cloning. The analysis of genes related to reprogramming failure (Group 2–3) using a PPI network revealed six HUB genes: *DDX55*, *EXOSC10*, *KRR1*, *MRPL4*, *PES1*, and *RPS13*. All these genes are related to ribosome and RNA metabolism. Incomplete reprogramming of rDNA after somatic cell nuclear transplantation has been observed in mice. The activity of rDNA after nuclear transplantation is regulated by the activity of rDNA in the donor cells before nuclear transplantation [36]. It has also been suggested that transient inhibition of rDNA in donor cells benefits the development of nuclear transplanted embryos [37]. It remains to be determined whether this approach can effectively address ribosomal pathway disorders in UC-MSCs when used as donor cells in sheep cloning.

The next analysis focused on chromosome-level expression. The results indicate that there was no skewing of chromosome-level expression on autosomes when UC-MSCs were used as donor nuclei. However, when fibroblasts were used as donor nuclei relative to IVF, most autosomes, particularly chromosome 4, showed skewed expression (Figure 9A). Additionally, both donor nuclei showed a down-regulation of chromosome-scale expression on the X chromosome. Previous studies have shown that abnormal X-chromosome reprogramming occurs in bovine [38], mouse [39], porcine [40], and buffalo [41] embryos. This is mainly caused by a disruption of *Xist* expression. However, it is still necessary to investigate whether *Xist* is responsible for X-chromosome expression disruption in sheep. Finally, the analysis of enzymes related to histone lysine methylation and DNA methylation revealed that although UC-MSCs could rescue most of the histone abnormalities, some abnormalities still existed in the H3K27, H3K4, H3K9, and H4K20 loci.

## 5. Conclusions

The RNA profiles of in vitro-fertilized eight-cell embryos and handmade-cloned sheep eight-cell embryos with umbilical cord mesenchymal stem cells and fibroblasts as donor nuclei were mapped and compared at the chromatin level, the level of differentially expressed genes, the level of histone modifications, and the level of DNA methylation. The results showed that the RNA profile of umbilical cord MSCs as donor nuclei was very similar to that of IVF embryos compared to fibroblasts as donor nuclei. However, the ribosomal pathway and histone loci H3K27, H3K4, H3K9, and H4K20 remained abnormal. This single-cell profile provides important information for using UC-MSCs as a preferred donor nucleus for nuclear transfer techniques, and also provides some unique clues for studying cell reprogramming mechanisms.

## Figures and Tables

**Table 1:** Information on RT-qPCR primer.

Genes	NCBI Accession Number	Primer Sequence (5′-3′)	Size (bp)	Tm (°C)
PCNA	XM_004014340.5	F: GAGGGCTTCGACACTTACCG R: TGCCAAGGTGTCCGCATTAT	138	60
POT1	XM_060415094.1	F: TGAAGTGGTTACGAGCAGTAAG R: CAACCTGGCTGTAGGGATCT	175	58
RUNX2	XM_027959124.1	F: GAGCAGGCAAGTTCCAACAGG R: ACGCAGTAGTAGACACCAGATTCC	140	58
LUM	XM_012174076.3	F: CTTAGACAACAACAAGATTAGCAACATCC R: GACTCCACTATCAGCCAGTTCATTATG	106	58
SOX9	XM_027974011.1	F: GCAGAAGGCAAGCAAAGGAGAC R: AGGTGATGGTGTTAGTGAGAGGAC	143	58
PPARG	NM_001100921.1	F: GCAGGAGCCCAGCAAAGAGG R: GTCATTCAAGTCAAGGTTCACAAAGC	130	58
PES1	XM_042234838.2	F: TTCCGGGAGTACAAGGTGTT R: ACGATGTGGTCGAGCTTGTA	116	59
DNASE1L1	XM_004022654.4	F: TACGTGTATCTCTACCGGTCAC R: AACCAGCACACAAAAGGCTC	101	59
ACTB	XM_060405599.1	F: CCCTGGAGAAGAGCTACGAG R: GGTAGTTTCGTGAATGCCGC	131	59

**Table 2:** Statistical results of RNA-seq mapped.

Library	Total Reads	Mapped Reads	Mapping Rate	MultiMap Reads	MultiMap Rate
DFF1	38,862,746	37,319,939	0.9655	1,917,676	0.0493
DFF2	40,358,892	39,113,777	0.9733	2,002,014	0.0496
DFF3	38,648,926	37,036,828	0.9648	1,915,352	0.0496
DFF4	49,298,014	47,440,125	0.9663	2,447,206	0.0496
DFUC1	40,024,928	38,664,225	0.9708	1,846,722	0.0461
DFUC2	41,473,274	40,099,748	0.9709	2,136,130	0.0515
DFUC3	43,030,326	41,303,712	0.9641	2,164,632	0.0503
DFUC4	42,487,408	40,218,212	0.9517	2,315,488	0.0545
IVF1	40,445,670	38,827,017	0.9653	2,693,004	0.0666
IVF2	39,362,696	37,653,823	0.9622	2,638,940	0.0670
IVF3	44,804,330	43,255,434	0.9697	2,202,814	0.0492
IVF4	40,421,886	39,202,553	0.9738	2,098,936	0.0519

## Data Availability

The data presented in this study are available on request from the corresponding author.
